# Prevalence and predictors of bullying among in-school adolescents in Nigeria

**DOI:** 10.1016/j.jtumed.2023.05.009

**Published:** 2023-05-23

**Authors:** Isabella G. Ighaede-Edwards, Xiaoqun Liu, David B. Olawade, Jonathan Ling, Aderonke Odetayo, Aanuoluwapo C. David-Olawade

**Affiliations:** a Department of Maternal and Child Health, Xiangya School of Public Health, Central South University, Changsha, Hunan, China; b Center for Population and Reproductive Health, University of Ibadan, Nigeria; c Faculty of Life Sciences and Education, University of South Wales, Wales, United Kingdom; d Faculty of Health Sciences and Wellbeing, University of Sunderland, United Kingdom; e School of Public Health, The University of Hong Kong, SAR, Hong Kong; f Endoscopy Unit, Epsom and St Helier University Hospitals, NHS Trust, United Kingdom

**Keywords:** التنمر, المراهق, التنبؤ, المدارس الثانوية, نيجيريا, Adolescent, Bullying, Nigeria, Predictors, Secondary schools

## Abstract

**Objective:**

As an emerging significant public health issue affecting many students globally, school bullying is a threat that should not be disregarded. While several published studies have focused on bullying in developed countries, very little is known about the prevalence and predictors of bullying in Nigeria. This study aimed to determine the prevalence and predictors of bullying in secondary schools in Edo State, Nigeria.

**Method:**

A descriptive cross-sectional study was conducted with 621 in-school adolescents using a multistage random sampling technique. The 40-item Olweus Bully/Victim Questionnaire (OBVQ) was utilized for data collection. The chi-squared test, Fisher's test, and binomial logistic regression analysis were utilized to examine associations between variables at 5% level of significance.

**Results:**

Approximately half of the respondents (51.9%) had experienced at least one type of bullying, while 173 (27.9%) reported themselves as bullies. The most common type of bullying was physical bullying in different forms (belonging taken/stolen-68.3%; kicked, pushed or locked indoor-52.2%; threatened-47.8%), while the most common location of bullying was the classroom in the absence of a teacher (75%); the perpetrators were reported by the majority (58.3%) to be classmates. Respondents in junior classes were 1.61-fold more likely to be bullied than those in senior classes (adjusted odds ratio [AOR]: 1.60; confidence interval [CI]: 1.15–2.24), those who live in rural areas were 1.75-fold more likely to be bullied than urban cities (AOR: 0.45; CI: 0.58–1.80), and those who were frequently beaten by their parents were 2.28-fold more likely to be bullies than those who were not beaten (AOR: 2.16; CI: 1.33–3.52). Furthermore, the act of bullying others was significantly associated with family monthly income (p = 0.01).

**Conclusion:**

Owing to the prevalence and predictors of bullying reported in this study, we recommend that policies should be implemented in schools to protect the most affected and vulnerable groups from being victims of school bullying.

## المخلص

**أهداف البحث:**

نظرا لكونه مشكلة صحية عامة ناشئة تؤثر على العديد من الطلاب على مستوى العالم ، فإن التنمر في المدارس يمثل تهديدا لا ينبغي تجاهله. في حين أن العديد من الدراسات المنشورة قد ركزت على التنمر في البلدان المتقدمة، إلا أن الدراسة الاستقصائية لانتشار التنمر والتنبؤ به في نيجيريا لم تخضع للبحث الكافي. هدفت هذه الدراسة إلى تحديد مدى انتشار التنمر والتنبؤ به في المدارس الثانوية في ولاية إيدو، نيجيريا.

**طرق البحث:**

أجريت دراسة مقطعية وصفية على 621 مراهقا في المدرسة باستخدام تقنية أخذ العينات العشوائية متعددة المراحل. تم استخدام استبيان "أولويس التنمر/الضحية" المكون من 40 عنصرا لجمع البيانات. تم استخدام اختبار مربع كاي، واختبار فيشر، وتحليل الانحدار اللوجستي ذي الحدين لفحص الارتباطات بين المتغيرات عند مستوى الأهمية 5 ٪.

**النتائج:**

حوالي نصف المستطلعين (51.9٪) تعرضوا لنوع واحد على الأقل من التنمر، بينما أفاد 173 (27.9٪) بأنهم متنمرون. كان أكثر أنواع التنمر شيوعا هو التنمر الجسدي بأشكال مختلفة، بينما كان المكان الأكثر شيوعا للتنمر هو الفصل في غياب المعلم (75٪)، وتم الإبلاغ عن الجناة من قبل الغالبية (58.3٪) من زملاء الدراسة. كان المستجيبون في الصفوف الإعدادية 1.61 أكثر عرضة للتخويف من أولئك الموجودين في الصفوف العليا، أولئك الذين يعيشون في المناطق الريفية 1,75 أكثر احتمالية من المدن الحضرية، وأولئك الذين تعرضوا للضرب بشكل متكرر من قبل آبائهم كانوا 2,28 أكثر عرضة من غيرهم. أيضا، كان فعل التنمر على الآخرين مرتبطا بشكل هام بالدخل الشهري للعائلة.

**الاستنتاجات:**

نظرا لانتشار التنمر والمتنبأ به المبلغ عنه في هذه الدراسة، نوصي بضرورة تنفيذ السياسات في المدارس لحماية الفئات الأكثر تضررا وضعفا من الوقوع ضحايا التنمر في المدرسة.

## Introduction

Bullying is a growing problem for adolescents globally, with many adverse short-term and long-term effects.^1^ Bullying is categorized into physical bullying, verbal bullying, relational bullying (rumor spreading and social exclusion), and cyberbullying. The increased use of computers and cell phones has exacerbated cyberbullying, particularly among teenagers.^2^ Bullying is regarded as a serious public health problem which can increase the risk of depression and anti-social behavior later in life.^3^^,^^4^

Bullying is any deliberate and persistent act of aggressive behavior against an individual carried out by a single person or a group of people where the victim has difficulty defending themselves due to an imbalance of power.^5^ Bullying affects a significant proportion of students daily in schools directly or indirectly, either as bullies, victims, or both.^1^^,^^3^^,^^5^, ^6^, ^7^ Bullying has been linked to a wide range of unfavorable outcomes, including poor academic performance, lower academic self-perceptions, poor school engagement, mental health issues, and negative behaviors that last into adulthood.^6^, ^7^, ^8^, ^9^, ^10^

Globally, peer-to-peer violence in and around schools is reportedly experienced by approximately 150 million students between the ages of 13 and 15 years, and around one in three students in this age group experience bullying that results in physical fights.^11^^,^^12^ A study across the six World Health Organization (WHO) regions found that bullying among adolescents was most prevalent in the Eastern Mediterranean (45.1%) and African regions (43.5%) and least prevalent in Europe (8.4%).^12^ In addition, data from the International Health Behavior of School Children survey reported that Sub-Saharan Africa (SSA) (48%), North Africa (43%), and the Middle East (41%) were the regions with the highest prevalence of school bullying.^13^^,^^14^

Previous studies have found that male gender, lower socioeconomic status, and younger age were linked with higher rates of bullying resulting in physical fights, while unfavorable rumors and loneliness were more common in females.^12^^,^^15^, ^16^, ^17^, ^18^ A study across the Organization of Economic Cooperation and Development (OECD) countries found that, on average, around 11% of students were being made fun of frequently, 8% were the target of nasty rumors, 7% reported being frequently excluded from activities, and 7.7% experienced occasional physical bullying.^16^

Despite the fact that bullying occurs globally, the majority of studies on this subject have been conducted in developed nations.^7^^,^^16^ According to available research, the prevalence of bullying in adolescents in Africa ranges between 16% and 63%.^12^^,^^19^, ^20^, ^21^, ^22^, ^23^ SSA, Malawi, Ghana, Zambia, and Sierra Leone all reported high incidences of bullying among in-school adolescents at incidences of 44.5%, 40.1%, 62.8%, and 54.6%, respectively.^19^, ^20^, ^21^^,^^24^ Furthermore, interpersonal violence has been reported to be prevalent in 53.7% of in-school adolescents in SSA.^25^ An age less than 15 years, male gender, low socioeconomic background, and behavioral traits such as social isolation, a history of cigarette smoking, alcohol, and drug use, have all been identified as predictors of bullying in SSA countries.^17^^,^^19^^,^^21^^,^^24^ In addition, a lack of supportive peers or friends, negative emotions such as anxiety, sadness, low self-esteem, or suicidal thoughts, and a lack of strong parent-child relationships, have been reported as predictors of interpersonal violence among in-school adolescents.^23^^,^^25^

In Nigeria, bullying in schools is a major issue. In a nationwide study of school violence conducted by the Federal Ministry of Education in collaboration with UNICEF, it was discovered that physical violence accounted for 85% of the majority of victimization against in-school children, while psychological violence accounted for 50% of in-school children victimization.^34^ Furthermore, physical violence was more common in rural areas (90%) than urban areas (80%); and was more common in the southern region (90%) than in the northern region (70%), while psychological violence affected 38.7% of people in the north compared to 61% in the south.^34^, ^35^, ^36^ Unfortunately, most parents and educators tend to view this as a normal aspect that growing children must learn to cope with.^26^ The estimated prevalence of bullying in Nigeria varies between 21% and 82%.^27^, ^28^, ^29^, ^30^, ^31^ Being male, having previously used alcohol, tobacco, or cannabis, having been involved in cults or gangs, coming from a polygamous home, having less religious parents, not having a fulfilling relationship with teachers, and various other sociocultural factors, have all been associated with bullying in Nigerian students.^27^, ^28^, ^29^, ^30^, ^31^, ^32^ In addition, peer group influence and exposure to media were also found to be predictors of bullying among students.^33^

Despite the severe consequences and high prevalence of bullying across various nations, this issue remains under-researched in Africa and Nigeria. Although some researchers have reported the prevalence of bullying in schools from different states in Nigeria, this research was conducted almost a decade ago.^27^, ^28^, ^29^^,^^32^^,^^34^, ^35^, ^36^ This demonstrates that there is still a dearth of research regarding the prevalence of bullying among school adolescents. Furthermore, bullying in Nigerian schools has not been addressed, and anti-bullying programs have yet to be implemented. Knowledge of the risk factors associated with bullying and victimization among Nigerian school students is important for the implementation of preventive measures against bullying. As a result, it is essential to assess the trend of bullying prevalence from earlier studies while also providing information on current trends. Therefore, this study aimed to determine the prevalence and predictors of bullying in secondary schools in Edo State, Nigeria.

## Methods

### Study area and study population

This study was carried out in Benin City, Edo State, located in the South–South region of Nigeria. Benin City is the capital and largest city of Edo State, with a total population of 1,782,000 as of 2021. The city has three local government areas (LGAs): Egor, Ikpoba-Okha, and Oredo. This study was conducted in six randomly selected secondary schools from the three local government areas of the city. Six hundred and twenty-one adolescents attending secondary schools (both junior and senior) of mixed schools (day and boarding), government-owned public schools, and government-approved private school students participated in the study.

### Study design and sampling technique

The study was a descriptive cross-sectional study. A multistage random sampling technique was utilized to select eligible respondents for the study. In the first stage, a line list of all schools in the study location was obtained from the State Ministry of Education. Then, a simple random sampling technique (balloting) was used to select 18 schools across the 3 local government areas (6 schools from each LGA). In the second stage, the schools were stratified into public and private schools. From the 18 schools selected earlier, a simple sampling technique was used to select one private and one public school across the 3 LGAs, which made a total of 6 selected schools (3 private and 3 public schools represented from all the LGAs). In the final stage, a convenience sampling technique was used to select 621 students across the selected six schools.

### Data collection tool

#### Olweus Bully/Victim Questionnaire (OBVQ)

This study utilized the Olweus Bully/Victim Questionnaire (OBVQ) to analyze the bullying behavior of secondary school students in Nigeria. The OBVQ is one of the most extensively used questionnaires to measure the prevalence of bullying globally.^37^^,^^38^ This 40-item questionnaire provides students with a clear definition of bullying and contains three key characteristics: (1) intent to cause harm to another person; (2) repetitive behavior; and (3) power imbalance between the victim and the perpetrator.^39^ The questionnaire also includes questions about different types of bullying, including sexual bullying and cyberbullying. The frequency of bullying was measured on a Likert scale, with participants being asked how many times they had been bullied in the previous 12 months: ‘I haven't been bullied in the last 12 months', ‘it has only happened once or twice’, ‘two or three times a week’, ‘about once a week’, and ‘several times a week’. The Likert-type scale has six response options; (0 = never; 1 = once or twice in the previous 12 months; 2 = three to six times in the previous 12 months; 3 = once a week; 4 = many times a week). Categories 1, 2, 3, and 4 were measured as being a victim of bully, and being bullies, respectively. Also, all participants who reported to have been bullied or have ever bullied others at least once or twice in the previous 12 months were considered a victim, or a bully, respectively, while those who reported never to have been bullied or never bullied others were considered not to be a victim of bullying, and not a bully, respectively.^32^

### Data analysis

Statistical Package for Social Sciences (SPSS) version 25.0 (IBM, Chicago, IL, United States) was used to enter and analyze the data. For every variable considered in the analysis, frequencies and percentages were computed. Chi-squared analysis and Fisher's exact test were conducted to detect associations between the socio-demographics and the outcome measures of bullying and victimization. Binomial logistic regression was used to determine factors that independently predict the outcome. The binomial logistic regression analysis included socio-demographic variables with p < 0.05.

## Results

### Psycho-social and demographic characteristics of respondents

Among the 621 adolescents who completed the survey (see Table 1), more than half were female and attended public school. The majority were in junior secondary school and were from monogamous homes, and over half reported that they lived with their parents. A little below half lived in urban regions and most of the students were day-schooled. Almost all considered their school as safe and only a few had disabilities. The majority (67.5%) reported that they had a very good relationship with their parents; only 3.7% and 2.1% mentioned that a bad relationship existed between them and their class teacher and schoolmates, respectively.

**Table 1:** Psycho-social and demographic characteristics of respondents.

Variable	Frequency	%
Age group		
Below 18	593	95.5
18 and above	28	4.5
Gender		
Male	294	47.3
Female	327	52.7
Type of school		
Public	321	51.7
Private	300	48.3
Class of Respondent		
Junior	379	61.0
Senior	242	39.0
Family type		
Monogamous	520	83.7
Polygamous	101	16.3
Marital status of parent		
Married	533	85.8
Divorced/separated	48	7.7
Widowed	40	6.4
Who do you live with presently?		
Parents	364	58.6
Single parent	90	14.5
Grandparent	84	13.5
Others	83	13.4
Family's Monthly Income		
Minimum wage	100	16.1
Middle/average	131	21.1
High	105	16.9
I don't know	285	45.9
Place of residence		
Urban	291	46.9
Semi Urban	260	41.9
Rural	70	11.3
School type		
Day	536	86.3
Day and boarding	85	13.7
Type of student		
Day	581	93.6
Boarding	40	6.4
How safe is your school?		
Very safe	268	43.2
Safe	265	42.7
Somewhat safe	62	10.0
Unsafe	26	4.2
Any disabilities?		
Yes	60	9.7
No	561	90.3
Relationship between you and your parents		
Very good	419	67.5
Good	128	20.6
It's okay	56	9.0
Bad	18	2.9
Frequency of beating/scolding by parents		
Always	76	12.2
Sometimes	321	51.7
Rarely	134	21.6
Never	90	14.5
Relationship between you and class teacher		
Very good	167	26.9
Good	296	47.7
It's okay	135	21.7
Bad	23	3.7
Relationship between you and classmates		
Very good	255	41.1
Good	266	42.8
It's okay	87	14.0
Bad	13	2.1

### Prevalence of bullying (being a victim of bullying/been bullied) and bullying others

A total of 322 out of the total population of 621 reported being bullied in one way or another. This accounts for 51.9% of the respondents been bullied in this study. Table 2 shows the typology of bullying. Among those who had been bullied, physical bullying, such as being threatened by others (47.8%), having money or other things taken from them (68.3%), along with hitting, kicking, pushing, shoving, or being locked indoors (52.2%), were the most common experiences. On the other hand, a total of 173 respondents reported to have participated in bullying others, accounting for 27.9% of those bullying others among the respondents. Table 2 also shows the different bullying behaviors.

**Table 2:** Typology of bullying experienced and the frequency of bullying others.

Bullying typology	No	Yes	Only once or twice	2 or 3 times a month	About once a week	Several times a week
Called mean names, was made fun of, or teased in a hurtful way (verbal bullying)	101 (31.4)	221 (68.6)	130 (40.4)	32 (9.9)	25 (7.8)	34 (10.6)
Other students left me out or ignored me (relational bully)	137 (42.5)	185 (57.5)	126 (39.1)	35 (10.9)	14 (4.3)	10 (3.1)
Hitting, kicking, pushing, shoving around, or locked indoors (physical bully)	154 (47.8)	168 (52.2)	111 (34.5)	31 (9.6)	14 (4.3)	12 (3.7)
Told lies/spread rumors (relational bully)	131 (40.7)	191 (59.3)	125 (38.8)	28 (8.7)	16 (5.0)	22 (6.8)
Stolen, taken belongings (physical bully)	102 (31.7)	220 (68.3)	160 (49.7)	29 (9.0)	20 (6.2)	11 (3.4)
Threatened (physical bully)	168 (52.2)	154 (47.8)	124 (38.5)	18 (5.6)	8 (2.5)	4 (1.2)
Bullied due to tribe, size or religion or family (verbal bully)	177 (55.0)	145 (45.0)	95 (29.5)	22 (6.8)	10 (3.1)	18 (5.6)
Cyberbully	168 (52.2)	154 (47.8)	107 (33.2)	23 (7.1)	13 (4.0)	11 (3.4)
I was bullied in another way	223 (69.3)	99 (30.7)	64 (19.9)	22 (6.8)	4 (1.2)	9 (2.8)
**Frequency of bullying others**	**No**	**Yes**	**Only once or twice**	**2 or 3 times a month**	**About once a week**	**Several times a week**
I called another student(s) mean names, made fun of or teased him or her in a hurtful way	62 (35.8)	111 (64.2)	80 (46.2)	12 (6.9)	7 (4.0)	12 (6.9)
I kept him or her out of things on purpose, excluded him or her from my group of friends or completely ignored him or her	86 (49.7)	87 (50.3)	66 (38.2)	9 (5.2)	12 (6.9)	0 (0.)
I hit, kicked, pushed, and shoved him or her around or locked him or her indoors.	86 (49.7)	87 (50.3)	70 (40.5)	7 (4.0)	5 (2.9)	5 (2.9)
I spread false rumors about him or her and tried to make others dislike him or her.	110 (63.6)	63 (36.4)	38 (22.0)	13 (7.5)	10 (5.8)	2 (1.2)
I took money or other things from him or her or damaged his or her belongings.	116 (67.1)	57 (32.9)	39 (22.5)	7 (4.0)	2 (1.2)	9 (5.2)
I threatened or forced him or her to do things he or she didn't want to do.	98 (56.6)	75 (43.4)	58 (33.5)	6 (3.5)	4 (2.3)	7 (4.0)
I bullied him or her with mean names or comments about his or her tribe or size or religion or family.	98 (56.6)	75 (43.4)	58 (33.5)	2 (1.2)	8 (4.6)	7 (4.0)
I bullied him or her with mean or hurtful messages, calls, or pictures, or in other ways on my mobile phone or over the internet (computer).	122 (70.5)	51 (29.5)	31 (17.9)	15 (8.7)	3 (1.7)	2 (1.2)

### Location of bullying, reporting of bullying, and characteristics of the bullying

The most commonly reported location of bullying was in the classroom when teachers were not present (75%), followed by in the classroom when teachers were present (50.8%). Among those who had been bullied, and reported their bullying experiences, the class teacher (30.0%) and parent/guardian (30.5%) were the commonest responses. Among those who were bullied, the majority were bullied by a member of their class (58.3%); 31.1% were bullied by several boys and the majority (69.9%) said their bullying lasted for 1–2 weeks. Of those who were bullied, 122 (62.6%) reported when they had been bullied while 73 (38.4%) did not report their experience after been bullied (See Table 3).

**Table 3:** Location of bullying, reporting of bullying, and characteristics of bullying.

Location of bullying	Frequency	%
In the classroom (when the teacher was not present)	96	75.0
In the classroom (when the teacher was present)	65	50.8
On the playground/field (during recess or break times)	48	37.5
In the hallways/stairwells	47	36.7
^I^n the bathroom	44	34.4
On the way to and from school	39	30.5
In the school dormitory	39	30.5
At the school bus stop	37	28.9
On the school bus	37	28.9
In the dining hall/cafeteria	34	26.6
In the locker room	31	24.2
**Bullying reporting among respondent [Multiple response (N = 122)]**	**Frequency**	**%**
Class teacher	36	30.5
Parent/guardian	36	30.5
Friend	33	28.0
Brother	27	22.9
School principal	22	18.6
Other teacher	16	13.6
Someone else	12	10.2
**Characteristics of the bullying**	**Frequency**	**%**
Class of student who bullied you My class Different class but same grade (year) A higher grade A lower grade	105 27 29 19	58.3 15.0 16.1 10.6
Have you been bullied by boys or girls (n = 219) Mainly by a girl By several girls Mainly by a boy By several boys By both boys and girls	32 17 57 68 45	14.6 7.8 26.0 31.1 20.5
By how many students have you usually been bullied Mainly by one student By a group of 2–3 students By a group of 4–9 students By a group of more than 9 students	86 82 28 19	40.0 38.1 13.0 8.9
How long has the bullying lasted (n = 196) 1–2 weeks About a month About 6 months About a year and/or more	137 27 17 15	69.9 13.8 8.7 7.7

### Association between psycho-social and demographics and being a victim of bullying

Bivariate analysis with chi-squared and Fisher's Exact tests was applied where required due to small cell sizes; see Table 4); these analyses showed that the class of the respondents, place of residence, and the frequency of being beaten by a parent, were significantly associated with being a victim of bullying among the participants.

**Table 4:** Association between psycho-social and demographic characteristics and being a victim of bullying.

Variable	Victim		χ^2^	df	P-value
	No (%)	Yes (%)			
Age group					
Below 18	289 (48.7)	304 (51.3)	1.82	1	0.18
18 and above	10 (35.7)	18 (64.3)			
Gender					
Male	136 (46.3)	158 (53.7)	0.80	1	0.37
Female	163 (49.8)	164 (50.2)			
Type of school					
Public	156 (48.6)	165 (51.4)	0.82	1	0.82
Private	143 (47.7)	157 (52.3)			
Class of Respondent					
Junior	165 (43.5)	214 (56.5)	8.29	1	0.004^a^
Senior	134 (55.4)	108 (44.6)			
Family type					
Monogamous	259 (49.8)	261 (50.2)	3.53	1	0.06
Polygamous	40 (38.6)	61 (60.4)			
Marital status of parent					
Married	254 (47.7)	279 (52.3)	0.37	1	0.55
Divorced/separated/widowed/No parent	45 (51.1)	43 (48.9)			
Who do you live with presently?					
Parents	170 (46.7)	194 (53.3)	1.60	4	0.80
Single parent	44 (48.9)	46 (51.1)			
Grandparent	40 (47.6)	44 (52.4)			
	3 (50.0)	3 (50.0)			
	42 (54.5)	35 (45.5)			
Family's Monthly Income					
Minimum wage	39 (39.0)	61 (61.0)	4.10	3	0.25
Middle/average	67 (51.1)	64 (48.9)			
High	52 (49.5)	53 (50.5)			
I don't know	141 (49.5)	144 (50.5)			
Place of residence					
Urban	131 (45.0)	160 (55.0)	6.39	2	0.04^a^
Semi Urban	140 (53.8)	120 (46.2)			
Rural	28 (40.0)	42 (60.0)			
School type					
Day	261 (48.7)	275 (51.3)	0.47	1	0.49
Day and boarding	38 (44.7)	47 (55.3)			
Type of student					
Day	284 (48.9)	297 (51.1)	1.94	1	0.16
Boarding	15 (37.5)	25 (62.5)			
How safe is your school?					
Safe	284 (47.7)	311 (52.3)	0.99	1	0.32
Unsafe	15 (57.7)	11 (42.3)			
Any disabilities?					
Yes	26 (43.3)	34 (56.7)	0.62	1	0.43
No	273 (48.7)	288 (51.3)			
Relationship between you and your parents					
Good	289 (47.9)	314 (52.1)	0.41	1	0.52
Bad	10 (55.6)	8 (44.4)			
Frequency of beating/scolding by parents					
Always	35 (46.1)	41 (53.9)	18.59	3	<0.001^a^
Sometimes	131 (40.8)	190 (59.2)			
Rarely	78 (58.2)	56 (41.8)			
Never	55 (61.1)	35 (38.9)			
Relationship between you and class teacher					
Good	291 (48.7)	307 (51.3)	1.71	1	0.19
Bad	8 (34.8)	15 (65.2)			
Relationship between you and classmates					
Good	294 (48.4)	314 (51.6)	0.50	1	0.48
Bad	5 (38.5)	8 (61.5)			

a Significant.

Table 5 shows results from bivariate analysis with chi-square (and Fisher's Exact tests where required due to small cell sizes), number of siblings, family monthly income, were significantly associated with bullying others among the participants.

**Table 5:** Association between psycho-social and demographic characteristics and bullying others.

Variable	Bully		χ^2^	df	P value
	No (%)	Yes (%)			
Age group					
Below 18	427 (72.0)	166 (28.0)	0.12	1	0.73
18 and above	21 (75.0)	7 (25.0)			
Gender					
Male	202 (68.7)	92 (31.3)	3.28	1	0.07
Female	246 (75.2)	81 (24.8)			
Type of school					
Public	231 (72.0)	90 (28.0)	0.01	1	0.92
Private	217 (72.3)	83 (27.7)			
Class of Respondent					
Junior	272 (71.8)	107 (28.2)	0.07	1	0.80
Senior	176 (72.7)	66 (27.3)			
Family type					
Monogamous	378 (72.7)	142 (27.3)	0.48	1	0.49
Polygamous	70 (69.3)	31 (30.7)			
Marital status of parent					
Married	390 (73.2)	143 (26.8)	1.98	1	0.16
Divorced/separated/widowed/No parent	58 (65.9)	30 (34.1)			
Family's Monthly Income					
Minimum wage	64 (64.0)	36 (36.0)	10.85	3	0.01^a^
Middle/average	85 (64.9)	46 (35.1)			
High	79 (75.2)	26 (24.8)			
I don't know	220 (77.2)	65 (22.8)			
Place of residence					
Urban	217 (74.6)	74 (25.4)	2.42	2	0.30
Semi Urban	185 (71.2)	75 (28.8)			
Rural	46 (65.7)	24 (34.3)			
School type					
Day	416 (71.6)	165 (28.4)	1.31	1	0.25
Day and boarding	32 (80.0)	8 (20.0)			
Type of student					
Day	379 (70.7)	157 (29.3)	4.00	1	0.05
Boarding	69 (81.2)	16 (18.8)			
How safe is your school?					
Safe	429 (72.1)	166 (27.9)	0.01	1	0.91
Unsafe	19 (73.1)	7 (26.9)			
Any disabilities?					
Yes	43 (71.7)	17 (28.3)	0.01	1	0.93
No	405 (72.2)	156 (27.8)			
Relationship between you and your parents					
Good	435 (72.1)	168 (27.9)	0.00	1	0.99
Bad	13 (72.2)	5 (27.8)			
Frequency of beating/scolding by parents					
Always	57 (75.0)	19 (25.0)	1.75	3	0.63
Sometimes	233 (72.6)	88 (27.4)			
Rarely	98 (73.1)	36 (26.9)			
Never	60 (66.7)	30 (33.3)			
Relationship between you and class teacher					
Good	434 (72.6)	164 (27.4)	1.51	1	0.22
Bad	14 (60.9)	9 (39.1)			
Relationship between you and classmates					
Good	442 (72.7)	166 (27.3)	4.46	1	0.06
Bad	6 (46.2)	7 (53.8)			

a Significant.

### Factors associated with being victims of bullying

As shown in Table 6, results from univariate analysis with logistic regression showed that participants in Junior class were 1.61 times more likely to be bullied when compared to those in senior class while those living in a semi-urban region were 1.75 times less likely to be bullied when compared to those living in a rural community. Participants who reported that their parents sometimes beat them were 2.28 times more likely to be bullied when compared to those participants who said they were never beaten by a parent. Multivariate analysis showed that participants in Junior class were 1.60 times more likely to be bullied when compared to those in senior class. Those who were being beaten “always” and “sometimes” by a parent compared to not being beaten by parent were 1.97 times and 2.16 times more likely to be bullied.

**Table 6:** Factors associated with being victims of bullying and bullying others.

Factors associated with being victims of bullying						
Variables	OR	P value	95% CI	AOR	P value	95% CI
Class of Respondent						
Junior	1.61	0.004	1.16–2.23	1.60	0.01	1.15–2.24
Senior	1					
Place of residence						
Urban	0.81	0.45	0.81–0.48	1.03	0.93	0.58–1.80
Semi Urban	0.57	0.04	0.57–0.33	0.73	0.28	0.41–1.30
Rural	1					
Frequency of beating/scolding by parents						
Always	1.84	0.05	0.99–3.42	1.97	0.04	1.05–3.68
Sometimes	2.28	0.001	1.41–3.68	2.16	0.002	1.33–3.52
Rarely	1.13	0.67	0.65–1.95	1.07	0.81	0.62–1.86
Never	1					
**Factors associated with bullying others**						
	OR	P value	95% CI	AOR	P-value	95% CI
Gender						
Male	1.38	0.07	0.97–1.97	1.15	0.40	0.83–1.58
Female						
Do you have siblings?						
1	2.35	0.001	1.43–3.85	1.10	0.71	0.67–1.79
2	1.37	1.16	0.87–2.12	1.25	0.27	0.84–1.87
3 or more	1					
Family's Monthly Income						
Low	1	0.89	0.56–1.66	0.60	0.06	0.35–1.01
Middle/average	0.96	0.08	0.32–1.07	0.62	0.10	0.35–1.10
High	0.59	0.01	0.32–0.86	0.62	0.05	0.38–0.99
I don't know	0.53					
Type of student						
Day	1.59	0.26	0.72–3.52			
Boarding	1					
Relationship between you and classmates						
Good	0.32	0.04	0.11–0.97	0.74	0.62	0.23–2.39
Bad	1					

### Factors associated with bullying others

Results from univariate analysis with logistic regression showed that participants with only one sibling were 2.35 times more likely to bully others when compared to those with 3 or more siblings. Those who did not know their family monthly income were 1.88 times less likely to bully others when compared to those with a low income. Participants who had a good relationship with classmates were 3.13 times less likely to bully others when compared to those who had a bad relationship with class mates. There were no other significant variables (see Table 6).

## Discussion

The findings in this study showed that over half of the respondents (51.9%) had experienced bullying at least once in the past few months. This proportion is higher than results obtained from other African countries, including Malawi, Ghana, and South Africa, where the prevalence of bullying was reported to be 25%–50%.^19^^,^^20^^,^^22^^,^^23^ In the United States, bullying among students was reported to range from 16% to 45%, and from 6% to 8% among Canadian adolescents.^40^, ^41^, ^42^, ^43^, ^44^, ^45^, ^46^ This difference could be the result of positive measures, such as the school bullying prevention programs that advanced nations have taken to combat the threat of victimization in schools.^47^ Sierra Leone and Zambia had higher percentages of 54.6% and 62.8%, respectively, than that of our study, while Egypt also reported a very high bullying prevalence of 77.8%.^21^^,^^24^^,^^48^ Furthermore, the prevalence of bullying in this study was comparable to studies reported in Osun (50%), Ondo (27.5%), and Oyo (47.9%),^28^^,^^35^^,^^49^ but was relatively low compared to studies conducted in Benin City (80%), Sokoto (65%), and Port Harcourt (82.2%) states in Nigeria.^27^^,^^30^^,^^32^ Physical bullying was the most common type in this population because this is seen as either a sign of older students demanding respect from younger ones or the intimidation of peers to instill fear. Furthermore, children experience some sort of bullying from home as a sign of correction from parents or from school as corporal punishment. In addition, students who are bullies were identified as victims of bullying, either by older peers or parents.

The most common type of bullying reported in this study was physical bullying, such as hitting, kicking, or shoving, stealing belongings, or threats. This finding is consistent with previous work conducted in Nigeria and other low-and-middle-income countries.^12^^,^^15^^,^^28^^,^^30^^,^^34^^,^^35^^,^^50^ Another common type of bullying experienced in this study was verbal bullying, such as hurtful name-calling or making fun of tribes or families, followed by relational bullying, such as being ignored or spreading rumors, then cyberbullying. In contrast to physical bullying, verbal bullying was reported to be more common in some studies.^32^^,^^51^ The prevalence of verbal bullying in some schools may be because school policies expressly forbid physical fighting.^52^ Furthermore, the effects of verbal bullying are often emotional, resulting in low self-esteem or, in some cases, depression.^53^^,^^54^

In this study, 27.9% of students participated in bullying others, which means that a larger proportion (72.1%) were victims of bullying. This corresponds with other studies, where the proportion of bully victims outweighed bullies.^16^^,^^18^, ^19^, ^20^^,^^26^, ^27^, ^28^^,^^34^^,^^55^ Over half of the students (58.3%) in our study were bullied by their classmates, who were mostly boys. This agrees with other studies in that boys are more likely to be bullies and victims than girls.^23^^,^^24^^,^^26^^,^^31^^,^^35^^,^^49^ Bullying was related to location; a large proportion of respondents (75%) reported being bullied in the classroom without teachers being present, while the least reported location for bullying was in the locker room (24.2%).

Our study showed that over half (62.6%) of bullying victims reported their experiences to someone, while 38.4% did not report to anyone. The proportion of students who did not report their experience of bullying experience was similar to that reported by previous studies.^58^ Parents and class teachers were the most common individuals to receive reports of bullying from those affected. This may be because parents are perceived by their children as caretakers, and teachers are represented in schools as guardians. However, the proportion of those who reported bullying to their parents or teachers in this study was still quite low (30.5% and 30%, respectively). This indicates that parents and teachers ought to collaborate to tackle the problem of bullying, and schools to foster a supportive atmosphere where students feel free to open up about their experiences with bullying.

In this study, more males than females were victims of bullying (53.7% vs 50.2%), and more males were bullies (31.3% vs 24.3%). This finding resonates with earlier studies, which reported that boys are more likely than girls to be bullies, and also more likely to be victims.^32^^,^^35^^,^^45^^,^^49^^,^^56^^,^^57^ However, findings from South-west (Osun State), Nigeria and South-east (Anambra and Enugu States), Nigeria reported that girls bully more than boys, while other studies reported that girls are more likely to be victims than boys.^44^^,^^55^^,^^59^^,^^60^ This might be because of the higher prevalence of relational and cyberbullying among girls.^16^, ^17^, ^18^^,^^28^^,^^43^^,^^61^^,^^62^

Our findings revealed a significant association between psycho-social and demographic characteristics and being a victim of bullying. Students in the junior class were at higher risk of being victims of bullying than students in the senior class. Children who lived in rural areas were bullied more than those in urban areas, and children who experienced occasional scolding or beating from parents were twice as likely to be bully victims. In contrast, students from low-income families were more likely to bully other students. This correlates with several previous studies that reported that an age younger than 18 years, being in a junior class, coming from a low socio-economic class, and several behavioral traits, are all associated with the prevalence of being a victim of bullying.^12^^,^^15^^,^^17^^,^^19^, ^20^, ^21^, ^22^, ^23^, ^24^, ^25^, ^26^^,^^29^^,^^30^^,^^35^^,^^63^, ^64^, ^65^ We believe that there is a significant association between psychosocial and demographic characteristics in relation to bullying because, in Nigeria, low socio-economic families go through more financial hardship, which in turn affects the children, making them feel insecure and vulnerable in school and among their peers, thereby leading to social isolation and negative emotions such as anxiety. Furthermore, students in junior classes are most likely to be bullied due to their younger age, lack of friends or peers, low self-confidence, and poor social skills.

Furthermore, students who have a good relationship with their classmates are three times less likely to be bullies or victims. This is similar to previous studies, which reported that a lack of supportive peers or students wanting to establish dominance among new peers were predictors of bullying.^23^^,^^25^^,^^35^^,^^64^ This shows that adolescents from low social backgrounds are likely to have fewer friends, and those who have friends are more susceptible to peer pressure, thus indirectly impacting their decisions to either be bullies or bully victims. Hence, bullying prevention programs in schools should focus on students of younger age and in junior classes because these groups are more likely to be victims (or perpetrators) of bullying.

We are aware of limitations in this study. First, because our survey featured a cross-sectional design, it is impossible to draw conclusions on the cause-and-effect relationships on bullying prevalence. Second, although bullying was prevalent in this study, the participants self-reported their experiences of bullying; furthermore, recruitment was performed by convenience sampling. Thus, we cannot completely rule out recall bias. Finally, participants attended mixed (day and boarding) government-owned public schools and government-approved private schools, which may differ in terms of bullying experience and self-reporting. The strength of this study is that it is consistent with several other studies carried out in Africa,^19^^,^^20^^,^^22^^,^^23^ the United States and Canada,^40^, ^41^, ^42^, ^43^, ^44^, ^45^, ^46^ and OECD countries^16^ that clearly demonstrates that the prevalence of bullying among adolescents is a public health issue that needs urgent attention.

## Conclusion and recommendations

Bullying in adolescence is a significant public health issue that can have a long-lasting impact even after the adolescent years have passed. This study informs on the recent prevalence of bullying among in-school adolescents and policies to mitigate impact. However, to our knowledge, most studies conducted on bullying and anti-bullying programs in Nigeria were performed almost a decade ago. Therefore, research must be used to consistently assess the prevalence of bullying and the effectiveness of anti-bullying campaigns.

This study provides knowledge to caregivers (parents, guardians, teachers, schools, health care providers), school administration, health organizations, and government authorities, to help target anti-bullying strategies and policies. Our findings will help to understand the factors influencing bullying and the negative impacts and outcomes of bullying on society at large to help improve the school environment and make it safer for all learners. Parents should teach their children empathy and kindness towards others. Teachers and school administrators should work to ensure that bullying is not tolerated. The government should work together with school administration to launch anti-bullying campaigns to foster an environment in the classroom that is favorable to teaching and learning. School healthcare workers and counselors should coordinate with school administrators to arrange seminars on bullying, its effects, and ways to stop it.

## Source of funding

This research received no specific grant from any funding agency, commercial or not-for-profit sectors.

## Conflicts of interest

The authors report no conflicts of financial or non-financial interest in this work.

## Ethical approval and informed consent

The authors assert that all procedures contributing to this work comply with the ethical standards of the relevant national and institutional committees on human experimentation and with the Helsinki Declaration of 1975, as revised in 2008. The study was approved by the Research Ethics Committee of University of Benin, Edo State, Nigeria, and administrative approval was obtained from the schools’ principals. Also, informed consent was obtained from all individual participants included in the study.

## Authors' contributions

IGI conceived and designed the study, provided the research materials, reviewed, and edited the final draft of the article. ZL supervised the study process, reviewed the first draft of the article, and provided logistic support. DBO conducted the research, provided logistic support, analyzed, and interpreted data. JL provided research materials, reviewed, and edited the final draft of the article. AO wrote initial and final draft of the article. ACD collected and entered the data. All authors have critically reviewed and approved the final draft and are responsible for the content and similarity index of the manuscript.

## Data sharing statement

The data that support the findings of this study are available upon request from the corresponding author.
